# Lean mass reference curves in adolescents using dual-energy x-ray absorptiometry (DXA)

**DOI:** 10.1371/journal.pone.0228646

**Published:** 2020-02-06

**Authors:** Wagner Luis Ripka, Camila E. Orsso, Andrea M. Haqq, Thais Gretis Luz, Carla M. Prado, Leandra Ulbricht

**Affiliations:** 1 Graduate Program in Biomedical Engineering, Universidade Tecnológica Federal do Paraná, Curitiba, Brazil; 2 Department of Agricultural, Food and Nutritional Science, Li Ka Shing Centre for Health and Research, University of Alberta, Edmonton, Canada; 3 Department of Pediatrics, University of Alberta, Edmonton, Canada; University of Extremadura, SPAIN

## Abstract

The body composition phenotype of low lean mass (LM) has been associated with metabolic disorders and impaired physical functioning in the pediatric population. Abnormalities in body composition may be identified using reference curves; however, no reference data on LM is available for Brazilian adolescents. The purpose of this study was to present reference data, including percentile curves, of whole body LM, lean mass index (LMI), appendicular lean mass (ALM), and fat mass for Southern Brazilian adolescents. This was a cross-sectional study of adolescents aged 12–17 years from a southern region in Brazil, who had body composition assessed using dual energy x-ray absorptiometry (DXA). Percentile values and reference curves employing the Lambda, Mu and Sigma method (LMS) were computed for LM, LMI (lean mass/height^2^), ALM and fat mass. Data on 541 adolescents (68.6% boys) was included. Sex differences in growth trajectories were observed for absolute and adjusted LM, with boys presenting greater LM quantity with advancing ages than girls (66.9% and 17.4% difference between the ages of 12 and 17 for boys and girls, respectively). The values corresponding to the lowest percentile (3^rd^) of LMI ranged between 10.63 to 13.93 kg/m^2^ in boys and 11.13 to 12.03 kg/m^2^ among girls aged 12–17 years. This study established the first LM, LMI, and ALM reference curves in Southern Brazilian adolescents, which can potentially be used in association with functional measures to identify LM abnormalities during growth.

## Introduction

The recognition of sarcopenia as a condition associated only with aging is obsolete [1]. Several studies report that children and adolescents may also exhibit a body composition phenotype of low lean mass and strength [2,3]. Lean mass is the body compartment mainly composed of skeletal muscle, excluding fat and bone masses. Although the excess fat mass in the pediatric population continues to be a challenging public health issue in both developed and developing countries [4], low lean mass may also represent a significant burden to health-care system [1]. This condition has been shown to affect locomotion, posture, and metabolism in children and adolescents [2,5,6]. In fact, boys and girls with lean mass below the 25^th^ percentile had 21.2 and 3.61 times greater risk of metabolic syndrome, respectively, than those with lean mass above the 25^th^ percentile [7].

Reference data is necessary, as a first step, to identify individuals with low lean mass across the age spectrum. Emerging research has quantified lean mass in the pediatric population for the development of lean mass reference curves using dual energy x-ray absorptiometry (DXA), a reference body composition technique that is relatively simple and of minimal burden to the individual [8,9]. Studies conducted in several countries including the United States [10], Canada [11], UK [12], China [13], India [14], and Korea [2] employed the DXA technique, providing reference data for lean mass in children and adolescents. A similar pattern of lean mass accrual was seen across studies; although lean mass increased with age in both sexes, boys had a greater accretion of this body compartment than girls. Nevertheless, as sociodemographic, genetic and lifestyle factors influence body composition, population-specific data is valuable to reduce the risk of misclassifying the low lean mass phenotype among adolescents.

The amount of lean mass in children and adolescents from developing countries, such as Brazil, may differ from those of other countries. Brazil is a continental size country and the fifth most populous in the world and to our knowledge no studies have quantified lean mass of Brazilian adolescents. Therefore, the aim of this study was to present percentile reference values for whole body lean mass, lean mass index (LMI), appendicular lean mass (ALM), and fat mass in Southern Brazilians aged 12 to 17 years old.

## Methods

### Study population

This was a cross-sectional study of adolescents, aged of 12 to 17 years, recruited at public/private schools or recreational sports programs between 2014 to 2016 in Curitiba and its metropolitan region (composed of 29 other municipalities), state of Paraná, Brazil. After receiving information about the study through a presentation by the research team, those adolescents who showed interest to participate were included if meeting the following criteria: a) parents authorized their participation; b) did not take calcium medication; c) did not undergo radiography or computed tomography seven days prior to the evaluation; d) did not suspect pregnancy (self-reported). To ensure that the data was representative of the geographic region, the required sample size was estimated assuming a sampling error of 4.5%, specified at 91% confidence level of a universe of 82,414 individuals [4]. The study was conducted at *Universidade Tecnológica Federal do Paraná*, after parents signed a consent form. The study protocol was approved through the *Plataforma Brasil* system (protocol number: 11583113.7.0000.5547).

### Anthropometric and body composition evaluation

Anthropometric measurements included height and weight. Height was measured to the nearest 0.1 cm with a portable stadiometer with participants standing in bare feet. Body mass was assessed using a mechanical scale.

Body composition (lean mass, and fat mass) was assessed using whole body fan beam DXA scan, software version 13.3.0.1 (Hologic Discovery A, Hologic Inc., Bedford, USA). DXA was calibrated daily and all scans were performed by a single technician, according to manufacturer’s instructions. The coefficient of variation for DXA scan using the manufacturer's phantom was lower than 2%. Coefficient of variation above 2% precluded data acquisition and a new calibration was needed. Appendicular lean mass was calculated as the sum of lean mass from arms and legs. Fat mass, lean mass, and ALM are presented as absolute values (in kg) and as percentage of body weight. Lean mass index was calculated as lean mass divided by squared height (kg/m^2^).

### Data analysis

The normality of data distribution was initially evaluated using the Kolmogorov-Smirnov test within each sex and age categories. Continuous variables were presented as median and interquartile range (IQR) stratified by sex and age groups. Interquartile range was calculated as the difference between the third (i.e. upper quartile) and the first (i.e. lower quartile). To test for differences between age groups, one-way analysis of variance (ANOVA) was used followed by the Tukey post-hoc test. The Kruskal-Wallis test was applied to non-parametric data. A p<0.05 was considered statistically significant.

The construction of percentiles was performed using the Lambda, Mu and Sigma method (LMS) developed by Cole and Green [15], where L is the skewness, M represents the median, and S is the coefficient of variation. Centiles are computed by using the values of the LMS parameters to a given age with the following equation:

$$Measurement\;percentile=M(1+L\times S\times z_{\alpha }{)}^{\frac{1}{L}}$$ (1)

Where: z_α_ is the α-th centile to the distribution. The statistical analysis and further chart generation were performed using the LMS Chartmaker Pro Version 2.54 software program (Cambrige, UK) [15]. We included in the analysis smoothed LMS curves for the 3^rd^, 10^th^, 25^th^, 50^th^, 75^th^, 90^th^ and 97^th^ percentiles of lean mass, ALM, LMI, and fat mass.

## Results

Data on 541 adolescents was included (68.8% boys). Descriptive analyses of body composition, stratified by age, are shown in Tables 1 (boys) and 2 (girls). Whole body lean mass increased more than 66% in boys and 17% in girls between ages 12 and 17 years. Differences between age categories were observed in most variables, except for fat mass (in kg) for boys, and fat mass (in %) and ALM for girls. Post-hoc analysis is presented in S1 and S2 Tables.

**Table 1:** Descriptive anthropometric and body composition characteristics by age categories in boys (median [interquartile range]).

Parameters/Age	12	13	14	15	16	17	Total
N	33	45	87	73	74	59	371
Weight (kg)*	43.70 (16.35)	54.00 (16.75)	57.70 (15.20)	59.40 (12.80)	61.85 (10.13)	65.00 (12.10)	59.40 (14.20)
Height (m)*	1.52 (0.13)	1.61 (0.12)	1.67 (0.10)	1.71 (0.08)	1.73 (0.08)	1.74 (0.09)	1.69 (0.11)
BMI (kg/m^2^)*	18.60 (6.65)	19.78 (4.53)	20.62 (4.34)	20.69 (3.28)	20.90 (3.09)	21.91 (3.52)	20.78 (3.72)
FM (kg)	11.24 (9.78)	11.46 (6.50)	11.17 (3.76)	10.70 (4.03)	10.64 (3.27)	11.77 (4.24)	10.97 (4.45)
FM (%)*	25.40 (13.00)	22.20 (6.45)	18.30 (4.60)	17.80 (5.35)	17.30 (2.78)	17.50 (4.50)	18.50 (5.70)
LM (kg)*	31.42 (8.45)	38.49 (13.27)	44.59 (11.40)	46.98 (7.69)	49.18 (8.09)	52.44 (7.80)	46.18 (10.92)
LM (%)*	71.40 (11.15)	75.00 (5.15)	78.30 (4.50)	78.90 (4.90)	79.45 (3.93)	79.30 (4.70)	78.30 (5.70)
ALM (kg)*	13.18 (3.75)	16.72 (6.36)	20.02 (5.12)	21.05 (3.41)	22.28 (4.28)	22.90 (4.11)	20.54 (5.29)
ALM (%)*	29.90 (5.35)	33.40 (3.70)	35.10 (3.40)	34.70 (4.45)	35.85 (3.95)	34.90 (3.50)	34.70 (4.10)
LMI (kg/m^2^)*	13.51 (3.21)	14.24 (3.00)	15.93 (2.82)	16.24 (2.34)	16.27 (8.45)	17.49 (2.58)	16.07 (2.74)
BMC (kg)*	1.37 (0.33)	1.63 (0.52)	2.03 (0.58)	2.20 (0.46)	2.40 (0.46)	2.54 (0.58)	2.15 (0.75)

Appendicular lean mass (ALM); body mass index (BMI); bone mineral content (BMC); difference between ages (*); fat mass (FM); lean mass (LM); lean mass index (LMI).

**Table 2:** Descriptive anthropometric and body composition characteristics according to age categories in girls (median [interquartile range]).

Parameters/Age	12	13	14	15	16	17	Total
N	26	23	22	31	41	27	170
Weight (kg)*	50.65 (16.95)	52.00 (12.50)	55.25 (13.48)	52.00 (12.00)	59.60 (19.75)	60.90 (15.60)	55.15 (14.23)
Height (m)*	1.53 (0.11)	1.58 (0.10)	1.59 (0.12)	1.61 (0.09)	1.61 (0.10)	1.60 (0.09)	1.59 (0.11)
BMI (kg/m^2^)*	21.08 (6.17)	20.23 (17.12)	22.09 (7.25)	20.16 (4.61)	23.71 (5.57)	24.27 (5.02)	21.40 (5.64)
FM (kg)*	16.24 (10.21)	16.93 (6.99)	17.90 (8.21)	16.26 (9.78)	21.16 (9.71)	20.48 (9.11)	18.51 (8.64)
FM (%)	31.85 (7.70)	31.40 (5.70)	32.40 (6.53)	32.10 (9.00)	36.10 (7.30)	34.20 (8.40)	33.45 (7.20)
LM (kg)*	32.86 (6.44)	34.12 (5.27)	35.05 (7.78)	35.49 (6.34)	36.36 (10.10)	38.58 (5.85)	35.68 (7.50)
LM (%)	65.65 (7.45)	65.60 (7.40)	64.15 (6.53)	64.60 (9.80)	61.50 (8.45)	62.70 (7.90)	63.90 (7.60)
ALM (kg)	13.55 (2.85)	14.35 (2.81)	14.13 (3.82)	14.63 (3.10)	15.12 (4.51)	15.58 (2.86)	14.70 (3.31)
ALM (%)	26.80 (4.48)	27.60 (4.10)	26.35 (3.68)	27.70 (2.80)	26.30 (4.55)	25.00 (4.30)	26.80 (3.83)
LMI (kg/m^2^)*	13.64 (2.19)	13.76 (2.09)	14.43 (3.31)	13.56 (2.73)	14.40 (2.63)	15.24 (2.97)	13.93 (2.43)
BMC (kg)*	1.49 (0.41)	1.72 (0.33)	1.84 (0.49)	1.75 (0.35)	1.94 (0.43)	2.01 (0.29)	1.81 (0.37)

Appendicular lean mass (ALM); body mass index (BMI); bone mineral content (BMC); difference between ages (*); fat mass (FM); lean mass (LM); lean mass index (LMI).

**Fig 1:**
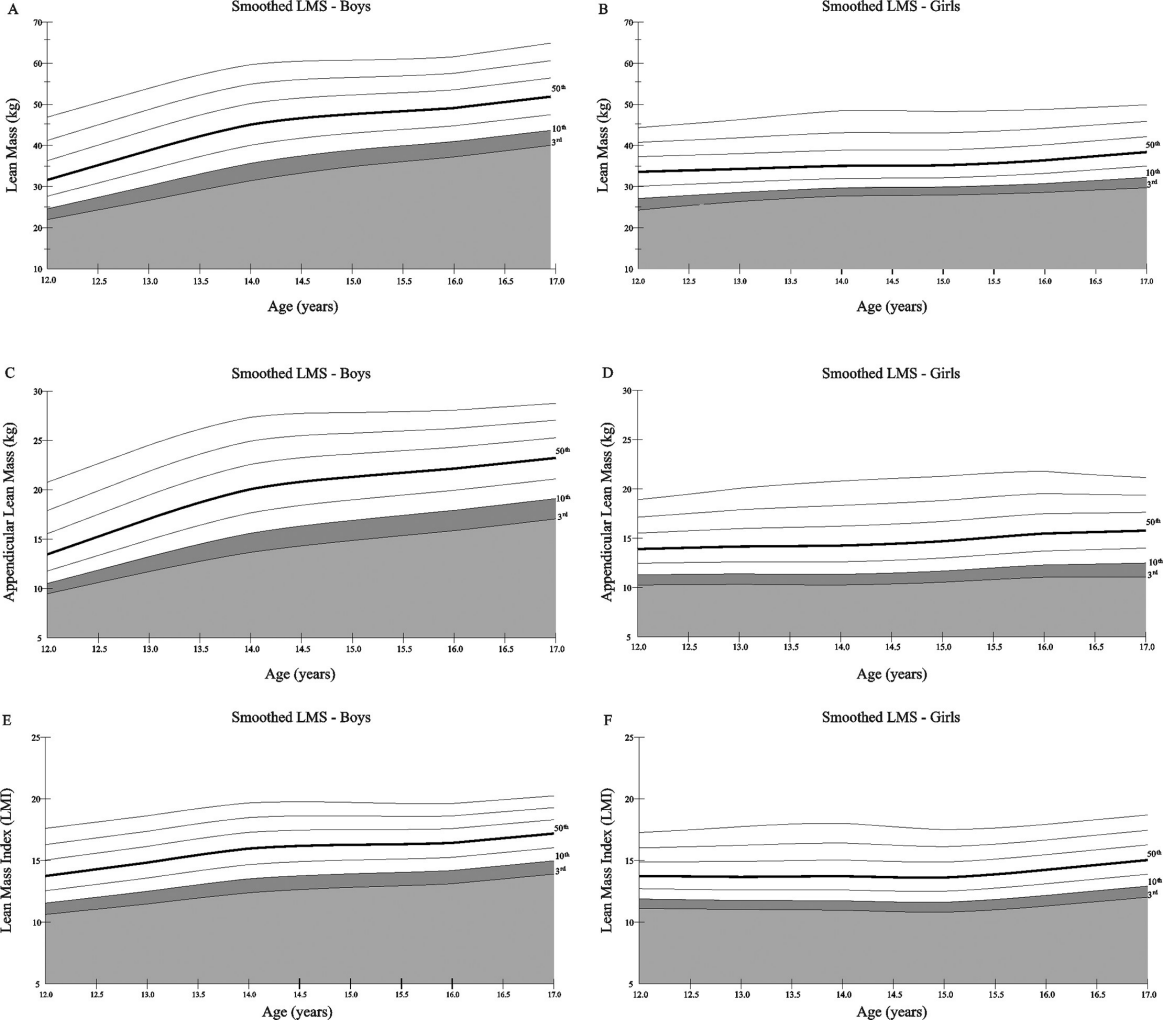
Smoothed LMS percentile curves for lean mass (A and B), appendicular lean mass (C and D) and lean mass index (E and F) in boys and girls. Curves represent the 3^rd^, 10^th^, 25^th^, 50^th^, 75^th^, 90^th^ and 97^th^ percentiles. Appendicular lean mass (ALM); lean mass (LM); lean mass index (LMI).

The smoothed age-specific percentiles and the corresponding parameters for lean mass (kg), ALM (kg) and LMI (kg/m^2^) are shown in Fig 1. LMS values for all age groups are presented in S3 and S4 Tables. For boys, lean mass varied gradually during adolescence with a 50^th^ percentile of 31.42 (IQR = 8.63) kg at age 12 and 52.44 (IQR = 8.79) kg at age 17, representing a 66.9% higher quantity of lean mass at older age. Conversely, the 50^th^ percentile for girls had values ranging from 32.86 (IQR = 7.20) kg (age 12) to 38.58 (IQR = 7.15) kg (age 17), approximately 17.4% higher at older age. Regarding LMI, the values corresponding to the lowest percentile (3^rd^) ranged between 10.63 to 13.93 kg/m^2^ in boys and 11.13 to 12.03 kg/m^2^ in girls aged 12–17 years.

**Fig 2:**
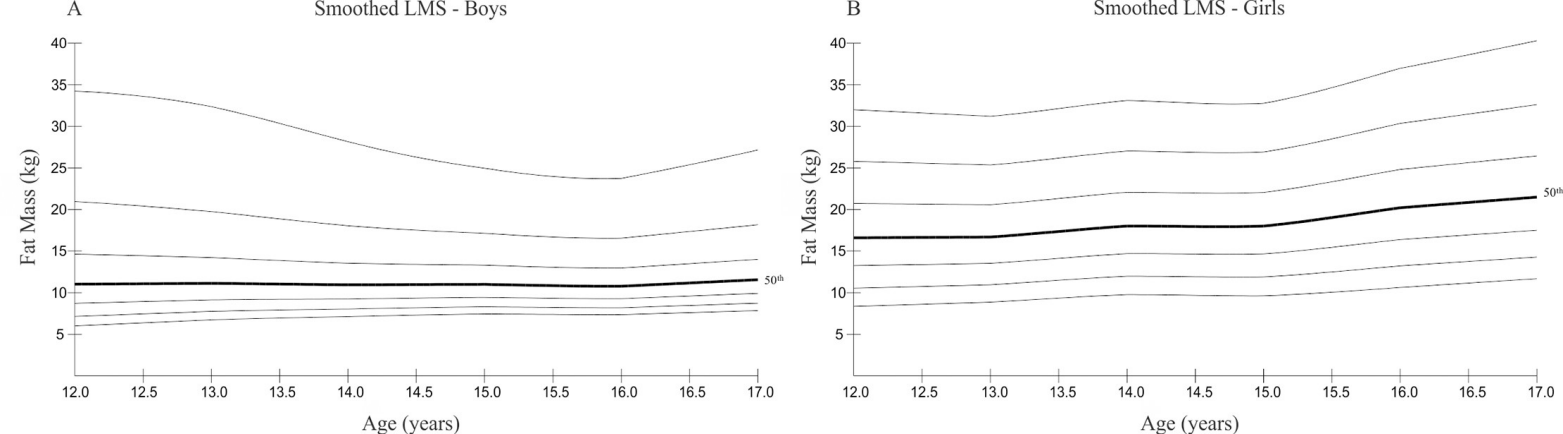
Smoothed LMS percentile curves for total fat mass (FM) for boys (A) and girls (B). Curves are for 3^rd^, 10^th^, 25^th^, 50^th^, 75^th^, 90^th^ and 97^th^ percentiles.

Reference curves for fat mass are shown in Fig 2. Boys in the 97^th^ percentile aged 16 years exhibited a lower content of fat mass compared to 12-year-old boys in the same percentile. In contrast, higher values of fat mass were observed with increasing age in girls. The 50^th^ percentile, for example, was 16.60 kg at the age of 12 years and 21.50 kg at the age of 17 years. Higher values were also seen in the 97^th^ percentile; girls exhibited 30.78 kg of fat mass at age 12 and 38.79 kg at the age 17.

**Fig 3:**
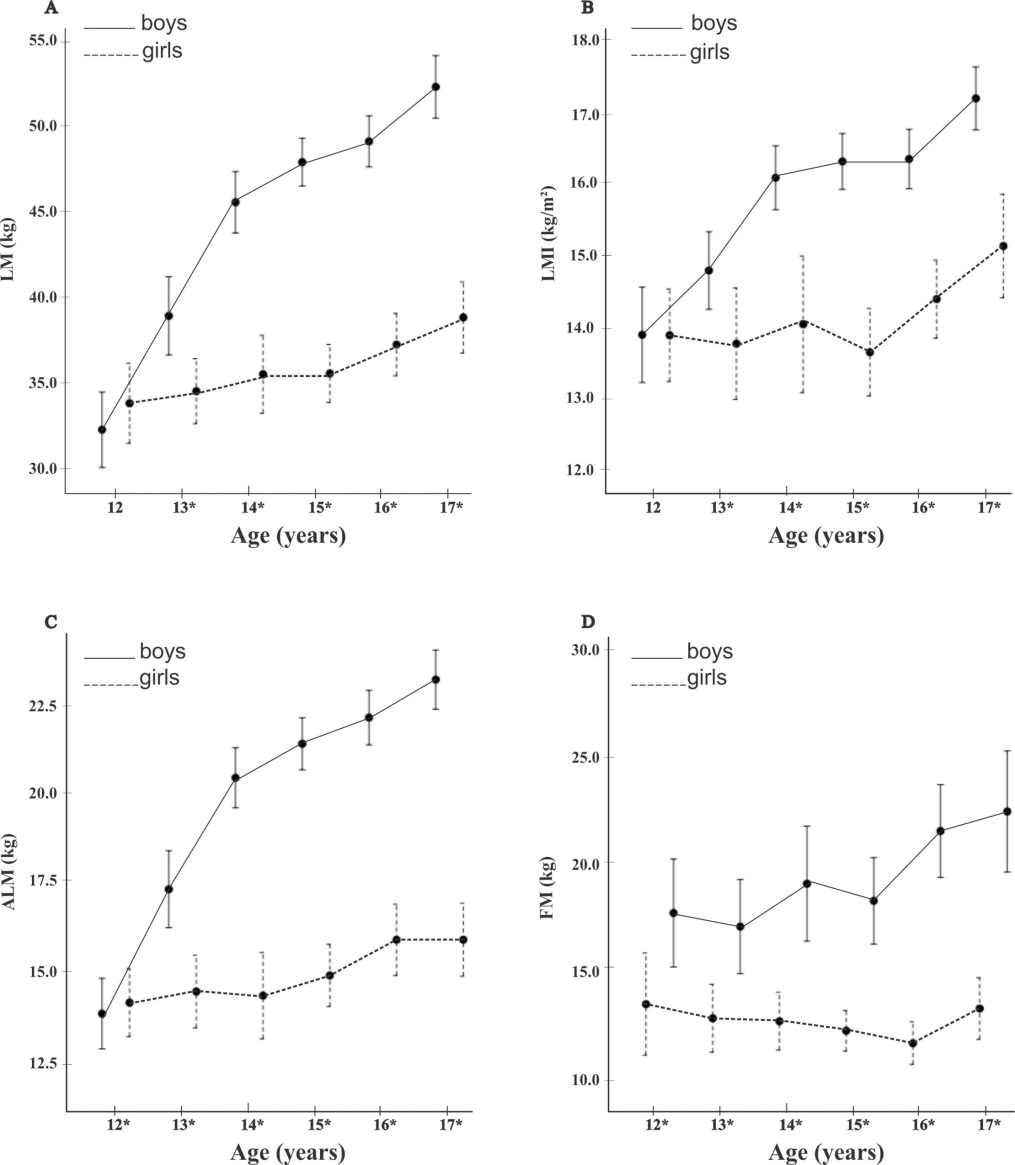
Age increments for (A) Lean Mass (LM) in kg, (B) Lean Mass Index (LMI) in kg/m^2^, (C) Appendicular Lean Mass (ALM) in kg and (D) fat mass (FM) in kg. Comparison for the boys and girls. (*) statistical difference between sex.

A comparison between sexes shown in Fig 3. Differences body composition variables were observed for all ages (mean with 95% confidence interval), except for those in the 12-year-old group (see S5 Table for p-values).

## Discussion

We present the first lean mass reference data for Southern Brazilian adolescents using DXA. Althought there is no established cut-off for defining low muscle mass in the pediatric population, the lowest LMI percentiles computed in our study (3^rd^ and 10^th^ percentile) could be used as an indicative of the low lean mass phenotype. Recently, Wells [16] described that body composition charts are major facilitators for monitoring of noncommunicable disease especially in low- and middle-income countries, strengthening the relevance of our study.

Differences in LMI between the lowest (3^rd^) and middle percentiles (50^th^) were approximately 3 kg/m^2^ in boys and girls. As the growth rate of lean mass peaks during adolescence, tracking its development in the pediatric population can lead to tailored nutritional and exercise interventions to improve treatment of metabolic diseases associated with sarcopenia in early in life [8,17]. Future studies combining these percentiles with functional data (e.g. muscle strength) are required for defining sarcopenia among children and adolescents [18].

As expected, absolute and adjusted values of lean mass for body size were higher with increasing age in boys and girls, but to a greater extent in boys. We also observed differing body composition between sexes in adolescents above age 13 years. This pattern of lean mass quantity and sexual dimorphism during adolescence is in agreement with previous findings from ethnically diverse populations and can be explained by hormonal influences [17,19–21]. A dramatic activation of growth hormone and sex steroids occur during pubertal growth spurt, which leads to rapid increases in skeletal muscle and, consequently, lean mass [22,23]. Although these hormonal changes occur in both sexes, girls enter puberty at an earlier chronological age than boys, therefore explaining differences in lean mass accrual between sexes. This distinction is further exemplified in a Canadian study, which has shown that while 65% of skeletal muscle (estimated by DXA from lean mass) was developed around 11.5 years in girls, 61% of the skeletal muscle was developed around the age of 13.7 years in boys [11]. Regarding fat mass, girls exhibited higher values with increasing ages than boys as they are expected to gain more fat than lean mass. This developmental pattern agrees with the literature on pediatric body composition and could be explained not only by sexual maturation but also lifestyle influences [24]; for example, girls had greater sedentary and physical inactivity behaviors than boys, as reported in our previous publication with the sample [4]. Thus, the percentile curves hereby computed for lean mass and fat mass had a similar shape to that of previously published charts during adolescence [2,20,21]. Despite finding similar patterns of lean mass quantity during adolescence to already established norms, comparisons with previously published data indicate substantial populational variability in lean mass. Our studied population from Southern Brazil exhibited greater lean mass (absolute and adjusted values) for each age group within sexes than adolescents from China [13], Thailand [19] and the United States [25] (exception for 16 years-old-boys and 15 years-old-girls); all studies used the same body composition technique (i.e. DXA). In contrast, a study conducted in the Netherlands reported that adolescents of both sexes aged 12 to 17 years had greater lean mass than adolescents from our study [17]. Moreover, compared to a study conducted in Korea, we found similarities in ALM among boys but a greater absolute amount of this body component in girls [2]. It could be argued that differences in lean mass across studies are mainly due to variations in ethnicity and sociodemographic and lifestyle factors in different countries [25].

These environmental factors also contribute to pubertal onset, which in turn lead to a differential development of lean mass [26]. As reported by Buyken et al [27], z-scores of fat-free mass adjusted for squared height (using skinfolds) were greater for adolescents who started pubertal growth spurt at an earlier chronological age than those with late puberty, independent of sex. We therefore speculate that differences in lean mass between our study and others could also be due to pubertal onset. Unfortunately, our study lacks data on sexual maturation and previous literature does not inform at which chronological age growth spurt occurs in Brazilian adolescents.

Nevertheless, these differences highlight the importance of developing population-specific reference data as using values from other countries would have led to inaccurate result for the evaluation of lean mass development in Brazilian adolescents. Likewise, it is unclear whether our charts are applicable to other geographic regions in Brazil given the diverse environment, which may influence dietary intake and physical activity patterns. The metropolitan area of Curitiba is among the ten largest population areas in Brazil. As most of the southern Brazilian population, Curitiba is inhabited mostly by people who are descendant from European cultures [4]. Thus, future multicenter studies should be conducted to evaluated whether adolescents from other Brazilian regions have similar lean mass.

Differences in body size among adolescents need to be accounted to present meaningful body composition data. Reporting lean mass and ALM data as percentage of body weight is incorrect (and meaningless), as changes in body fat could mask gains in lean mass during growth [8]. For example, although lean mass (in kg) increased in girls between ages 12 and 17 (p = 0.017), our data showed no differences in percentage lean mass between age groups (p = 0.470); the similarities in percentage lean mass with increasing age could be driven by accrual of fat mass instead (in kg; p = 0.036 between ages 13 to 17). An appropriate approach would be to correct measures of lean mass for height [8]. Despite the lack of evidence in adolescents, Wells and Cole [28] demonstrated that fat-free mass (as measured by deuterium dilution) should be adjusted for squared height in children specifically at the age of 8 years. More recently, a study from Peterson et al. using the NHANES database (n = 2285) indicated that body weight scaled more adequately to height cubed during puberty [29]. As this recent study [29] has not attempted to evaluate how lean mass scales to height yet, there is an urgent need to investigate this issue in future studies.

One limitation of our study is the lack of data on sexual maturation, limiting interpretation of the contribution of puberty to lean mass development. The development of our charts was also limited by the inability to use physical activity and dietary data as criteria for inclusion of participants, since these factors can have an impact on body composition. In spite of these limitations, the study certainly adds to our understanding of how lean mass differs across sexes and age categories in adolescents living in Southern Brazil. To measure lean mass accurately, we employed the DXA technique which is considered a method with high accuracy and reproducibility for body composition assessment for all age groups [30]. Despite greater cost than simpler techniques, DXA requires minimal participant compliance, offers minimal radiation exposure, and it is widely accepted as a measure of body composition during adolescence [30,31]. Furthermore, the LMS analysis is a popular method to obtain smoothened centile curves for cross-sectional data [32]. Thus, our charts are of great value to researchers studying adolescents from this geographical region and may be used by health care providers to identify abnormalities in body composition development during adolescence.

In conclusion, we presented age- and sex-specific reference data for lean and fat mass that are unique to Southern Brazilian adolescents. Although patterns of lean mass development were consistent with previous literature, researchers and health care providers cannot use these previous charts from other countries to monitor lean mass during adolescence in Southern Brazil because sociodemographic and lifestyle factors may affect body composition differently. It is also suggested future studies combining lean mass percentile data with handgrip strength measures from different regions in Brazil. This information would help the establishment of charts that can be used countrywide, identifying the risk of low lean mass and offering targeted treatments (e.g. nutritional and exercise interventions, and public policies for sport initiation in schools) to prevent associated outcomes such as sarcopenia.

## Supporting information

S1 Table Tukey pos-hoc for boys. (DOCX)

S2 Table Tukey pos-hoc for girls. (DOCX)

S3 Table LMS results for boys. (DOCX)

S4 Table LMS results for girls. (DOCX)

S5 Table Difference between sex for Lean Mass (LM). Appendicular Lean Mass (ALM). Lean Mass Index (LMI) and Fat Mass (FM).
(DOCX)
