# Supplementary material for: Lean mass reference curves in adolescents using dual-energy x-ray absorptiometry (DXA)
Source: PLoS One. 2020 Feb 6;15(2):e0228646. doi: 10.1371/journal.pone.0228646 (PMC7004364; doi:10.1371/journal.pone.0228646)
Supplement: S1 Table — (DOCX) [file pone.0228646.s001.docx]

**SUPPLEMENTARY MATERIAL**

| **Supplementary Table 1 - Tukey pos-hoc for Boys** | | | | | | | |
| --- | --- | --- | --- | --- | --- | --- | --- |
|  |  |  |  |  |  |  |  |
| **Multiple Comparisons - Boys** | | | | | | | |
| **Dependent Variable** | | | **Mean Difference (I-J)** | **Std. Error** | **Sig.** | **95% Confidence Interval** | |
|  |  |  |  |  |  | **Lower Bound** | **Upper Bound** |
| Weight (kg) | 12.00 | 13.00 | -6.00808 | 2.51007 | .161 | -13.1992 | 1.1831 |
|  |  | 14.00 | -12.49467^*^ | 2.23911 | .000 | -18.9096 | -6.0798 |
|  |  | 15.00 | -14.92756^*^ | 2.29740 | .000 | -21.5094 | -8.3457 |
|  |  | 16.00 | -15.81949^*^ | 2.29256 | .000 | -22.3875 | -9.2515 |
|  |  | 17.00 | -20.42013^*^ | 2.38074 | .000 | -27.2408 | -13.5995 |
|  | 13.00 | 12.00 | 6.00808 | 2.51007 | .161 | -1.1831 | 13.1992 |
|  |  | 14.00 | -6.48659^*^ | 2.01105 | .017 | -12.2481 | -.7251 |
|  |  | 15.00 | -8.91948^*^ | 2.07575 | .000 | -14.8664 | -2.9726 |
|  |  | 16.00 | -9.81141^*^ | 2.07039 | .000 | -15.7429 | -3.8799 |
|  |  | 17.00 | -14.41205^*^ | 2.16764 | .000 | -20.6222 | -8.2019 |
|  | 14.00 | 12.00 | 12.49467^*^ | 2.23911 | .000 | 6.0798 | 18.9096 |
|  |  | 13.00 | 6.48659^*^ | 2.01105 | .017 | .7251 | 12.2481 |
|  |  | 15.00 | -2.43289 | 1.73836 | .727 | -7.4132 | 2.5474 |
|  |  | 16.00 | -3.32482 | 1.73197 | .392 | -8.2868 | 1.6371 |
|  |  | 17.00 | -7.92546^*^ | 1.84711 | .000 | -13.2173 | -2.6336 |
|  | 15.00 | 12.00 | 14.92756^*^ | 2.29740 | .000 | 8.3457 | 21.5094 |
|  |  | 13.00 | 8.91948^*^ | 2.07575 | .000 | 2.9726 | 14.8664 |
|  |  | 14.00 | 2.43289 | 1.73836 | .727 | -2.5474 | 7.4132 |
|  |  | 16.00 | -.89193 | 1.80669 | .996 | -6.0680 | 4.2841 |
|  |  | 17.00 | -5.49257 | 1.91735 | .050 | -10.9856 | .0005 |
|  | 16.00 | 12.00 | 15.81949^*^ | 2.29256 | .000 | 9.2515 | 22.3875 |
|  |  | 13.00 | 9.81141^*^ | 2.07039 | .000 | 3.8799 | 15.7429 |
|  |  | 14.00 | 3.32482 | 1.73197 | .392 | -1.6371 | 8.2868 |
|  |  | 15.00 | .89193 | 1.80669 | .996 | -4.2841 | 6.0680 |
|  |  | 17.00 | -4.60064 | 1.91155 | .157 | -10.0771 | .8758 |
|  | 17.00 | 12.00 | 20.42013^*^ | 2.38074 | .000 | 13.5995 | 27.2408 |
|  |  | 13.00 | 14.41205^*^ | 2.16764 | .000 | 8.2019 | 20.6222 |
|  |  | 14.00 | 7.92546^*^ | 1.84711 | .000 | 2.6336 | 13.2173 |
|  |  | 15.00 | 5.49257 | 1.91735 | .050 | -.0005 | 10.9856 |
|  |  | 16.00 | 4.60064 | 1.91155 | .157 | -.8758 | 10.0771 |
| Height (m) | 12.00 | 13.00 | -.09461^*^ | .01596 | .000 | -.1403 | -.0489 |
|  |  | 14.00 | -.15520^*^ | .01424 | .000 | -.1960 | -.1144 |
|  |  | 15.00 | -.19184^*^ | .01461 | .000 | -.2337 | -.1500 |
|  |  | 16.00 | -.21182^*^ | .01458 | .000 | -.2536 | -.1701 |
|  |  | 17.00 | -.22128^*^ | .01514 | .000 | -.2647 | -.1779 |
|  | 13.00 | 12.00 | .09461^*^ | .01596 | .000 | .0489 | .1403 |
|  |  | 14.00 | -.06060^*^ | .01279 | .000 | -.0972 | -.0240 |
|  |  | 15.00 | -.09723^*^ | .01320 | .000 | -.1351 | -.0594 |
|  |  | 16.00 | -.11722^*^ | .01317 | .000 | -.1549 | -.0795 |
|  |  | 17.00 | -.12668^*^ | .01378 | .000 | -.1662 | -.0872 |
|  | 14.00 | 12.00 | .15520^*^ | .01424 | .000 | .1144 | .1960 |
|  |  | 13.00 | .06060^*^ | .01279 | .000 | .0240 | .0972 |
|  |  | 15.00 | -.03664^*^ | .01105 | .013 | -.0683 | -.0050 |
|  |  | 16.00 | -.05662^*^ | .01101 | .000 | -.0882 | -.0251 |
|  |  | 17.00 | -.06608^*^ | .01175 | .000 | -.0997 | -.0324 |
|  | 15.00 | 12.00 | .19184^*^ | .01461 | .000 | .1500 | .2337 |
|  |  | 13.00 | .09723^*^ | .01320 | .000 | .0594 | .1351 |
|  |  | 14.00 | .03664^*^ | .01105 | .013 | .0050 | .0683 |
|  |  | 16.00 | -.01998 | .01149 | .507 | -.0529 | .0129 |
|  |  | 17.00 | -.02945 | .01219 | .154 | -.0644 | .0055 |
|  | 16.00 | 12.00 | .21182^*^ | .01458 | .000 | .1701 | .2536 |
|  |  | 13.00 | .11722^*^ | .01317 | .000 | .0795 | .1549 |
|  |  | 14.00 | .05662^*^ | .01101 | .000 | .0251 | .0882 |
|  |  | 15.00 | .01998 | .01149 | .507 | -.0129 | .0529 |
|  |  | 17.00 | -.00946 | .01216 | .971 | -.0443 | .0254 |
|  | 17.00 | 12.00 | .22128^*^ | .01514 | .000 | .1779 | .2647 |
|  |  | 13.00 | .12668^*^ | .01378 | .000 | .0872 | .1662 |
|  |  | 14.00 | .06608^*^ | .01175 | .000 | .0324 | .0997 |
|  |  | 15.00 | .02945 | .01219 | .154 | -.0055 | .0644 |
|  |  | 16.00 | .00946 | .01216 | .971 | -.0254 | .0443 |
| BMI (kg/m²) | 12.00 | 13.00 | .02883 | .71959 | 1.000 | -2.0328 | 2.0904 |
|  |  | 14.00 | -.87480 | .64192 | .749 | -2.7138 | .9642 |
|  |  | 15.00 | -.91643 | .65862 | .732 | -2.8033 | .9705 |
|  |  | 16.00 | -.75259 | .65724 | .862 | -2.6355 | 1.1303 |
|  |  | 17.00 | -1.98510^*^ | .68252 | .044 | -3.9405 | -.0297 |
|  | 13.00 | 12.00 | -.02883 | .71959 | 1.000 | -2.0904 | 2.0328 |
|  |  | 14.00 | -.90362 | .57653 | .621 | -2.5554 | .7481 |
|  |  | 15.00 | -.94525 | .59508 | .607 | -2.6501 | .7596 |
|  |  | 16.00 | -.78142 | .59355 | .776 | -2.4819 | .9190 |
|  |  | 17.00 | -2.01393^*^ | .62142 | .016 | -3.7943 | -.2336 |
|  | 14.00 | 12.00 | .87480 | .64192 | .749 | -.9642 | 2.7138 |
|  |  | 13.00 | .90362 | .57653 | .621 | -.7481 | 2.5554 |
|  |  | 15.00 | -.04163 | .49836 | 1.000 | -1.4694 | 1.3861 |
|  |  | 16.00 | .12220 | .49652 | 1.000 | -1.3003 | 1.5447 |
|  |  | 17.00 | -1.11030 | .52953 | .291 | -2.6274 | .4068 |
|  | 15.00 | 12.00 | .91643 | .65862 | .732 | -.9705 | 2.8033 |
|  |  | 13.00 | .94525 | .59508 | .607 | -.7596 | 2.6501 |
|  |  | 14.00 | .04163 | .49836 | 1.000 | -1.3861 | 1.4694 |
|  |  | 16.00 | .16383 | .51795 | 1.000 | -1.3200 | 1.6477 |
|  |  | 17.00 | -1.06867 | .54967 | .377 | -2.6434 | .5061 |
|  | 16.00 | 12.00 | .75259 | .65724 | .862 | -1.1303 | 2.6355 |
|  |  | 13.00 | .78142 | .59355 | .776 | -.9190 | 2.4819 |
|  |  | 14.00 | -.12220 | .49652 | 1.000 | -1.5447 | 1.3003 |
|  |  | 15.00 | -.16383 | .51795 | 1.000 | -1.6477 | 1.3200 |
|  |  | 17.00 | -1.23251 | .54801 | .218 | -2.8025 | .3375 |
|  | 17.00 | 12.00 | 1.98510^*^ | .68252 | .044 | .0297 | 3.9405 |
|  |  | 13.00 | 2.01393^*^ | .62142 | .016 | .2336 | 3.7943 |
|  |  | 14.00 | 1.11030 | .52953 | .291 | -.4068 | 2.6274 |
|  |  | 15.00 | 1.06867 | .54967 | .377 | -.5061 | 2.6434 |
|  |  | 16.00 | 1.23251 | .54801 | .218 | -.3375 | 2.8025 |
| FM (%) | 12.00 | 13.00 | 3.64707^*^ | 1.15378 | .021 | .3416 | 6.9526 |
|  |  | 14.00 | 6.88370^*^ | 1.02923 | .000 | 3.9350 | 9.8324 |
|  |  | 15.00 | 7.89261^*^ | 1.05603 | .000 | 4.8672 | 10.9180 |
|  |  | 16.00 | 8.89746^*^ | 1.05380 | .000 | 5.8784 | 11.9165 |
|  |  | 17.00 | 8.09558^*^ | 1.09434 | .000 | 4.9604 | 11.2308 |
|  | 13.00 | 12.00 | -3.64707^*^ | 1.15378 | .021 | -6.9526 | -.3416 |
|  |  | 14.00 | 3.23663^*^ | .92440 | .007 | .5883 | 5.8850 |
|  |  | 15.00 | 4.24554^*^ | .95414 | .000 | 1.5120 | 6.9791 |
|  |  | 16.00 | 5.25039^*^ | .95168 | .000 | 2.5239 | 7.9769 |
|  |  | 17.00 | 4.44851^*^ | .99638 | .000 | 1.5940 | 7.3031 |
|  | 14.00 | 12.00 | -6.88370^*^ | 1.02923 | .000 | -9.8324 | -3.9350 |
|  |  | 13.00 | -3.23663^*^ | .92440 | .007 | -5.8850 | -.5883 |
|  |  | 15.00 | 1.00891 | .79906 | .805 | -1.2803 | 3.2982 |
|  |  | 16.00 | 2.01376 | .79612 | .118 | -.2671 | 4.2946 |
|  |  | 17.00 | 1.21188 | .84905 | .710 | -1.2206 | 3.6443 |
|  | 15.00 | 12.00 | -7.89261^*^ | 1.05603 | .000 | -10.9180 | -4.8672 |
|  |  | 13.00 | -4.24554^*^ | .95414 | .000 | -6.9791 | -1.5120 |
|  |  | 14.00 | -1.00891 | .79906 | .805 | -3.2982 | 1.2803 |
|  |  | 16.00 | 1.00485 | .83047 | .832 | -1.3744 | 3.3841 |
|  |  | 17.00 | .20297 | .88133 | 1.000 | -2.3220 | 2.7279 |
|  | 16.00 | 12.00 | -8.89746^*^ | 1.05380 | .000 | -11.9165 | -5.8784 |
|  |  | 13.00 | -5.25039^*^ | .95168 | .000 | -7.9769 | -2.5239 |
|  |  | 14.00 | -2.01376 | .79612 | .118 | -4.2946 | .2671 |
|  |  | 15.00 | -1.00485 | .83047 | .832 | -3.3841 | 1.3744 |
|  |  | 17.00 | -.80188 | .87867 | .943 | -3.3192 | 1.7154 |
|  | 17.00 | 12.00 | -8.09558^*^ | 1.09434 | .000 | -11.2308 | -4.9604 |
|  |  | 13.00 | -4.44851^*^ | .99638 | .000 | -7.3031 | -1.5940 |
|  |  | 14.00 | -1.21188 | .84905 | .710 | -3.6443 | 1.2206 |
|  |  | 15.00 | -.20297 | .88133 | 1.000 | -2.7279 | 2.3220 |
|  |  | 16.00 | .80188 | .87867 | .943 | -1.7154 | 3.3192 |
| FM (kg) | 12.00 | 13.00 | .66065 | 1.20913 | .994 | -2.8034 | 4.1247 |
|  |  | 14.00 | 1.00737 | 1.07861 | .938 | -2.0828 | 4.0975 |
|  |  | 15.00 | 1.24261 | 1.10668 | .872 | -1.9280 | 4.4132 |
|  |  | 16.00 | 1.82062 | 1.10435 | .567 | -1.3433 | 4.9845 |
|  |  | 17.00 | .20338 | 1.14683 | 1.000 | -3.0822 | 3.4890 |
|  | 13.00 | 12.00 | -.66065 | 1.20913 | .994 | -4.1247 | 2.8034 |
|  |  | 14.00 | .34672 | .96875 | .999 | -2.4287 | 3.1221 |
|  |  | 15.00 | .58196 | .99991 | .992 | -2.2827 | 3.4466 |
|  |  | 16.00 | 1.15998 | .99733 | .854 | -1.6973 | 4.0173 |
|  |  | 17.00 | -.45726 | 1.04418 | .998 | -3.4487 | 2.5342 |
|  | 14.00 | 12.00 | -1.00737 | 1.07861 | .938 | -4.0975 | 2.0828 |
|  |  | 13.00 | -.34672 | .96875 | .999 | -3.1221 | 2.4287 |
|  |  | 15.00 | .23524 | .83739 | 1.000 | -2.1638 | 2.6343 |
|  |  | 16.00 | .81326 | .83431 | .926 | -1.5770 | 3.2035 |
|  |  | 17.00 | -.80398 | .88977 | .945 | -3.3531 | 1.7452 |
|  | 15.00 | 12.00 | -1.24261 | 1.10668 | .872 | -4.4132 | 1.9280 |
|  |  | 13.00 | -.58196 | .99991 | .992 | -3.4466 | 2.2827 |
|  |  | 14.00 | -.23524 | .83739 | 1.000 | -2.6343 | 2.1638 |
|  |  | 16.00 | .57802 | .87030 | .986 | -1.9153 | 3.0714 |
|  |  | 17.00 | -1.03922 | .92361 | .871 | -3.6853 | 1.6069 |
|  | 16.00 | 12.00 | -1.82062 | 1.10435 | .567 | -4.9845 | 1.3433 |
|  |  | 13.00 | -1.15998 | .99733 | .854 | -4.0173 | 1.6973 |
|  |  | 14.00 | -.81326 | .83431 | .926 | -3.2035 | 1.5770 |
|  |  | 15.00 | -.57802 | .87030 | .986 | -3.0714 | 1.9153 |
|  |  | 17.00 | -1.61724 | .92082 | .495 | -4.2553 | 1.0208 |
|  | 17.00 | 12.00 | -.20338 | 1.14683 | 1.000 | -3.4890 | 3.0822 |
|  |  | 13.00 | .45726 | 1.04418 | .998 | -2.5342 | 3.4487 |
|  |  | 14.00 | .80398 | .88977 | .945 | -1.7452 | 3.3531 |
|  |  | 15.00 | 1.03922 | .92361 | .871 | -1.6069 | 3.6853 |
|  |  | 16.00 | 1.61724 | .92082 | .495 | -1.0208 | 4.2553 |
| LM (kg) | 12.00 | 13.00 | -6.63582^*^ | 1.61466 | .001 | -11.2617 | -2.0099 |
|  |  | 14.00 | -13.06481^*^ | 1.44036 | .000 | -17.1913 | -8.9383 |
|  |  | 15.00 | -15.59807^*^ | 1.47786 | .000 | -19.8320 | -11.3641 |
|  |  | 16.00 | -16.80646^*^ | 1.47474 | .000 | -21.0315 | -12.5814 |
|  |  | 17.00 | -19.98391^*^ | 1.53147 | .000 | -24.3715 | -15.5964 |
|  | 13.00 | 12.00 | 6.63582^*^ | 1.61466 | .001 | 2.0099 | 11.2617 |
|  |  | 14.00 | -6.42899^*^ | 1.29365 | .000 | -10.1352 | -2.7228 |
|  |  | 15.00 | -8.96226^*^ | 1.33527 | .000 | -12.7877 | -5.1368 |
|  |  | 16.00 | -10.17064^*^ | 1.33183 | .000 | -13.9862 | -6.3550 |
|  |  | 17.00 | -13.34809^*^ | 1.39438 | .000 | -17.3429 | -9.3533 |
|  | 14.00 | 12.00 | 13.06481^*^ | 1.44036 | .000 | 8.9383 | 17.1913 |
|  |  | 13.00 | 6.42899^*^ | 1.29365 | .000 | 2.7228 | 10.1352 |
|  |  | 15.00 | -2.53327 | 1.11824 | .211 | -5.7370 | .6704 |
|  |  | 16.00 | -3.74165^*^ | 1.11413 | .011 | -6.9335 | -.5498 |
|  |  | 17.00 | -6.91910^*^ | 1.18820 | .000 | -10.3232 | -3.5150 |
|  | 15.00 | 12.00 | 15.59807^*^ | 1.47786 | .000 | 11.3641 | 19.8320 |
|  |  | 13.00 | 8.96226^*^ | 1.33527 | .000 | 5.1368 | 12.7877 |
|  |  | 14.00 | 2.53327 | 1.11824 | .211 | -.6704 | 5.7370 |
|  |  | 16.00 | -1.20838 | 1.16219 | .904 | -4.5380 | 2.1212 |
|  |  | 17.00 | -4.38583^*^ | 1.23338 | .006 | -7.9194 | -.8523 |
|  | 16.00 | 12.00 | 16.80646^*^ | 1.47474 | .000 | 12.5814 | 21.0315 |
|  |  | 13.00 | 10.17064^*^ | 1.33183 | .000 | 6.3550 | 13.9862 |
|  |  | 14.00 | 3.74165^*^ | 1.11413 | .011 | .5498 | 6.9335 |
|  |  | 15.00 | 1.20838 | 1.16219 | .904 | -2.1212 | 4.5380 |
|  |  | 17.00 | -3.17745 | 1.22965 | .104 | -6.7003 | .3454 |
|  | 17.00 | 12.00 | 19.98391^*^ | 1.53147 | .000 | 15.5964 | 24.3715 |
|  |  | 13.00 | 13.34809^*^ | 1.39438 | .000 | 9.3533 | 17.3429 |
|  |  | 14.00 | 6.91910^*^ | 1.18820 | .000 | 3.5150 | 10.3232 |
|  |  | 15.00 | 4.38583^*^ | 1.23338 | .006 | .8523 | 7.9194 |
|  |  | 16.00 | 3.17745 | 1.22965 | .104 | -.3454 | 6.7003 |
| ALM (kg) | 12.00 | 13.00 | -3.48026^*^ | .77860 | .000 | -5.7109 | -1.2496 |
|  |  | 14.00 | -6.51665^*^ | .69455 | .000 | -8.5065 | -4.5268 |
|  |  | 15.00 | -7.54871^*^ | .71263 | .000 | -9.5904 | -5.5071 |
|  |  | 16.00 | -8.28272^*^ | .71113 | .000 | -10.3201 | -6.2454 |
|  |  | 17.00 | -9.34566^*^ | .73849 | .000 | -11.4614 | -7.2300 |
|  | 13.00 | 12.00 | 3.48026^*^ | .77860 | .000 | 1.2496 | 5.7109 |
|  |  | 14.00 | -3.03638^*^ | .62381 | .000 | -4.8236 | -1.2492 |
|  |  | 15.00 | -4.06845^*^ | .64388 | .000 | -5.9131 | -2.2238 |
|  |  | 16.00 | -4.80246^*^ | .64222 | .000 | -6.6424 | -2.9626 |
|  |  | 17.00 | -5.86540^*^ | .67238 | .000 | -7.7917 | -3.9391 |
|  | 14.00 | 12.00 | 6.51665^*^ | .69455 | .000 | 4.5268 | 8.5065 |
|  |  | 13.00 | 3.03638^*^ | .62381 | .000 | 1.2492 | 4.8236 |
|  |  | 15.00 | -1.03207 | .53922 | .395 | -2.5769 | .5128 |
|  |  | 16.00 | -1.76607^*^ | .53724 | .014 | -3.3052 | -.2269 |
|  |  | 17.00 | -2.82901^*^ | .57296 | .000 | -4.4705 | -1.1875 |
|  | 15.00 | 12.00 | 7.54871^*^ | .71263 | .000 | 5.5071 | 9.5904 |
|  |  | 13.00 | 4.06845^*^ | .64388 | .000 | 2.2238 | 5.9131 |
|  |  | 14.00 | 1.03207 | .53922 | .395 | -.5128 | 2.5769 |
|  |  | 16.00 | -.73401 | .56042 | .780 | -2.3396 | .8716 |
|  |  | 17.00 | -1.79695^*^ | .59474 | .032 | -3.5008 | -.0930 |
|  | 16.00 | 12.00 | 8.28272^*^ | .71113 | .000 | 6.2454 | 10.3201 |
|  |  | 13.00 | 4.80246^*^ | .64222 | .000 | 2.9626 | 6.6424 |
|  |  | 14.00 | 1.76607^*^ | .53724 | .014 | .2269 | 3.3052 |
|  |  | 15.00 | .73401 | .56042 | .780 | -.8716 | 2.3396 |
|  |  | 17.00 | -1.06294 | .59295 | .472 | -2.7617 | .6358 |
|  | 17.00 | 12.00 | 9.34566^*^ | .73849 | .000 | 7.2300 | 11.4614 |
|  |  | 13.00 | 5.86540^*^ | .67238 | .000 | 3.9391 | 7.7917 |
|  |  | 14.00 | 2.82901^*^ | .57296 | .000 | 1.1875 | 4.4705 |
|  |  | 15.00 | 1.79695^*^ | .59474 | .032 | .0930 | 3.5008 |
|  |  | 16.00 | 1.06294 | .59295 | .472 | -.6358 | 2.7617 |
| LMI (kg/m²) | 12.00 | 13.00 | -.90008 | .42210 | .273 | -2.1094 | .3092 |
|  |  | 14.00 | -2.16053^*^ | .37653 | .000 | -3.2393 | -1.0818 |
|  |  | 15.00 | -2.42505^*^ | .38633 | .000 | -3.5319 | -1.3182 |
|  |  | 16.00 | -2.46008^*^ | .38552 | .000 | -3.5646 | -1.3556 |
|  |  | 17.00 | -3.30629^*^ | .40035 | .000 | -4.4533 | -2.1593 |
|  | 13.00 | 12.00 | .90008 | .42210 | .273 | -.3092 | 2.1094 |
|  |  | 14.00 | -1.26045^*^ | .33818 | .003 | -2.2293 | -.2916 |
|  |  | 15.00 | -1.52497^*^ | .34906 | .000 | -2.5250 | -.5249 |
|  |  | 16.00 | -1.56000^*^ | .34816 | .000 | -2.5575 | -.5625 |
|  |  | 17.00 | -2.40621^*^ | .36451 | .000 | -3.4505 | -1.3619 |
|  | 14.00 | 12.00 | 2.16053^*^ | .37653 | .000 | 1.0818 | 3.2393 |
|  |  | 13.00 | 1.26045^*^ | .33818 | .003 | .2916 | 2.2293 |
|  |  | 15.00 | -.26452 | .29233 | .945 | -1.1020 | .5730 |
|  |  | 16.00 | -.29954 | .29125 | .908 | -1.1340 | .5349 |
|  |  | 17.00 | -1.14576^*^ | .31061 | .004 | -2.0356 | -.2559 |
|  | 15.00 | 12.00 | 2.42505^*^ | .38633 | .000 | 1.3182 | 3.5319 |
|  |  | 13.00 | 1.52497^*^ | .34906 | .000 | .5249 | 2.5250 |
|  |  | 14.00 | .26452 | .29233 | .945 | -.5730 | 1.1020 |
|  |  | 16.00 | -.03503 | .30382 | 1.000 | -.9054 | .8354 |
|  |  | 17.00 | -.88124 | .32243 | .071 | -1.8050 | .0425 |
|  | 16.00 | 12.00 | 2.46008^*^ | .38552 | .000 | 1.3556 | 3.5646 |
|  |  | 13.00 | 1.56000^*^ | .34816 | .000 | .5625 | 2.5575 |
|  |  | 14.00 | .29954 | .29125 | .908 | -.5349 | 1.1340 |
|  |  | 15.00 | .03503 | .30382 | 1.000 | -.8354 | .9054 |
|  |  | 17.00 | -.84621 | .32145 | .092 | -1.7671 | .0747 |
|  | 17.00 | 12.00 | 3.30629^*^ | .40035 | .000 | 2.1593 | 4.4533 |
|  |  | 13.00 | 2.40621^*^ | .36451 | .000 | 1.3619 | 3.4505 |
|  |  | 14.00 | 1.14576^*^ | .31061 | .004 | .2559 | 2.0356 |
|  |  | 15.00 | .88124 | .32243 | .071 | -.0425 | 1.8050 |
|  |  | 16.00 | .84621 | .32145 | .092 | -.0747 | 1.7671 |
| *. The mean difference is significant at the 0.05 level. | | | | | | | |
