# Supplementary material for: Lean mass reference curves in adolescents using dual-energy x-ray absorptiometry (DXA)
Source: PLoS One. 2020 Feb 6;15(2):e0228646. doi: 10.1371/journal.pone.0228646 (PMC7004364; doi:10.1371/journal.pone.0228646)
Supplement: S2 Table — (DOCX) [file pone.0228646.s002.docx]

| **SUPPLEMENTARY MATERIAL** | | | | | | | |
| --- | --- | --- | --- | --- | --- | --- | --- |
| **Supplementary Table 2 - Tukey pos-hoc for Girls** | | | | | | | |
|  |  |  |  |  |  |  |  |
| **Multiple Comparisons - Girls** | | | | | | | |
| **Dependent Variable** | | | **Mean Difference (I-J)** | **Std. Error** | **Sig.** | **95% Confidence Interval** | |
|  |  |  |  |  |  | **Lower Bound** | **Upper Bound** |
| Weight (kg) | 12.00 | 13.00 | -.15000 | 3.16641 | 1.000 | -9.2810 | 8.9810 |
|  |  | 14.00 | -5.18636 | 3.20437 | .588 | -14.4268 | 4.0541 |
|  |  | 15.00 | -2.51774 | 2.94164 | .956 | -11.0006 | 5.9651 |
|  |  | 16.00 | -7.56220 | 2.77318 | .075 | -15.5593 | .4349 |
|  |  | 17.00 | -10.08704^*^ | 3.03941 | .014 | -18.8518 | -1.3223 |
|  | 13.00 | 12.00 | .15000 | 3.16641 | 1.000 | -8.9810 | 9.2810 |
|  |  | 14.00 | -5.03636 | 3.29876 | .648 | -14.5490 | 4.4763 |
|  |  | 15.00 | -2.36774 | 3.04419 | .971 | -11.1463 | 6.4108 |
|  |  | 16.00 | -7.41220 | 2.88173 | .110 | -15.7223 | .8979 |
|  |  | 17.00 | -9.93704^*^ | 3.13877 | .022 | -18.9883 | -.8857 |
|  | 14.00 | 12.00 | 5.18636 | 3.20437 | .588 | -4.0541 | 14.4268 |
|  |  | 13.00 | 5.03636 | 3.29876 | .648 | -4.4763 | 14.5490 |
|  |  | 15.00 | 2.66862 | 3.08365 | .954 | -6.2237 | 11.5610 |
|  |  | 16.00 | -2.37583 | 2.92339 | .965 | -10.8060 | 6.0544 |
|  |  | 17.00 | -4.90067 | 3.17705 | .637 | -14.0624 | 4.2610 |
|  | 15.00 | 12.00 | 2.51774 | 2.94164 | .956 | -5.9651 | 11.0006 |
|  |  | 13.00 | 2.36774 | 3.04419 | .971 | -6.4108 | 11.1463 |
|  |  | 14.00 | -2.66862 | 3.08365 | .954 | -11.5610 | 6.2237 |
|  |  | 16.00 | -5.04445 | 2.63277 | .396 | -12.6366 | 2.5477 |
|  |  | 17.00 | -7.56930 | 2.91186 | .103 | -15.9663 | .8277 |
|  | 16.00 | 12.00 | 7.56220 | 2.77318 | .075 | -.4349 | 15.5593 |
|  |  | 13.00 | 7.41220 | 2.88173 | .110 | -.8979 | 15.7223 |
|  |  | 14.00 | 2.37583 | 2.92339 | .965 | -6.0544 | 10.8060 |
|  |  | 15.00 | 5.04445 | 2.63277 | .396 | -2.5477 | 12.6366 |
|  |  | 17.00 | -2.52484 | 2.74158 | .941 | -10.4308 | 5.3811 |
|  | 17.00 | 12.00 | 10.08704^*^ | 3.03941 | .014 | 1.3223 | 18.8518 |
|  |  | 13.00 | 9.93704^*^ | 3.13877 | .022 | .8857 | 18.9883 |
|  |  | 14.00 | 4.90067 | 3.17705 | .637 | -4.2610 | 14.0624 |
|  |  | 15.00 | 7.56930 | 2.91186 | .103 | -.8277 | 15.9663 |
|  |  | 16.00 | 2.52484 | 2.74158 | .941 | -5.3811 | 10.4308 |
| Height (m) | 12.00 | 13.00 | -.02896 | .01966 | .682 | -.0857 | .0277 |
|  |  | 14.00 | -.04643 | .01990 | .187 | -.1038 | .0109 |
|  |  | 15.00 | -.05881^*^ | .01827 | .019 | -.1115 | -.0061 |
|  |  | 16.00 | -.05047^*^ | .01722 | .044 | -.1001 | -.0008 |
|  |  | 17.00 | -.04536 | .01887 | .161 | -.0998 | .0091 |
|  | 13.00 | 12.00 | .02896 | .01966 | .682 | -.0277 | .0857 |
|  |  | 14.00 | -.01747 | .02049 | .957 | -.0765 | .0416 |
|  |  | 15.00 | -.02985 | .01890 | .614 | -.0844 | .0247 |
|  |  | 16.00 | -.02151 | .01790 | .836 | -.0731 | .0301 |
|  |  | 17.00 | -.01639 | .01949 | .959 | -.0726 | .0398 |
|  | 14.00 | 12.00 | .04643 | .01990 | .187 | -.0109 | .1038 |
|  |  | 13.00 | .01747 | .02049 | .957 | -.0416 | .0765 |
|  |  | 15.00 | -.01238 | .01915 | .987 | -.0676 | .0428 |
|  |  | 16.00 | -.00404 | .01815 | 1.000 | -.0564 | .0483 |
|  |  | 17.00 | .00108 | .01973 | 1.000 | -.0558 | .0580 |
|  | 15.00 | 12.00 | .05881^*^ | .01827 | .019 | .0061 | .1115 |
|  |  | 13.00 | .02985 | .01890 | .614 | -.0247 | .0844 |
|  |  | 14.00 | .01238 | .01915 | .987 | -.0428 | .0676 |
|  |  | 16.00 | .00834 | .01635 | .996 | -.0388 | .0555 |
|  |  | 17.00 | .01345 | .01808 | .976 | -.0387 | .0656 |
|  | 16.00 | 12.00 | .05047^*^ | .01722 | .044 | .0008 | .1001 |
|  |  | 13.00 | .02151 | .01790 | .836 | -.0301 | .0731 |
|  |  | 14.00 | .00404 | .01815 | 1.000 | -.0483 | .0564 |
|  |  | 15.00 | -.00834 | .01635 | .996 | -.0555 | .0388 |
|  |  | 17.00 | .00511 | .01703 | 1.000 | -.0440 | .0542 |
|  | 17.00 | 12.00 | .04536 | .01887 | .161 | -.0091 | .0998 |
|  |  | 13.00 | .01639 | .01949 | .959 | -.0398 | .0726 |
|  |  | 14.00 | -.00108 | .01973 | 1.000 | -.0580 | .0558 |
|  |  | 15.00 | -.01345 | .01808 | .976 | -.0656 | .0387 |
|  |  | 16.00 | -.00511 | .01703 | 1.000 | -.0542 | .0440 |
| BMI (kg/m²) | 12.00 | 13.00 | .51376 | 1.15970 | .998 | -2.8305 | 3.8580 |
|  |  | 14.00 | -1.83703 | 1.17360 | .623 | -5.2214 | 1.5473 |
|  |  | 15.00 | .35502 | 1.07738 | .999 | -2.7518 | 3.4619 |
|  |  | 16.00 | -1.67641 | 1.01568 | .566 | -4.6053 | 1.2525 |
|  |  | 17.00 | -2.88366 | 1.11318 | .105 | -6.0938 | .3264 |
|  | 13.00 | 12.00 | -.51376 | 1.15970 | .998 | -3.8580 | 2.8305 |
|  |  | 14.00 | -2.35079 | 1.20817 | .378 | -5.8348 | 1.1332 |
|  |  | 15.00 | -.15874 | 1.11494 | 1.000 | -3.3739 | 3.0564 |
|  |  | 16.00 | -2.19017 | 1.05544 | .305 | -5.2337 | .8534 |
|  |  | 17.00 | -3.39742^*^ | 1.14957 | .041 | -6.7125 | -.0824 |
|  | 14.00 | 12.00 | 1.83703 | 1.17360 | .623 | -1.5473 | 5.2214 |
|  |  | 13.00 | 2.35079 | 1.20817 | .378 | -1.1332 | 5.8348 |
|  |  | 15.00 | 2.19205 | 1.12939 | .381 | -1.0648 | 5.4489 |
|  |  | 16.00 | .16062 | 1.07069 | 1.000 | -2.9269 | 3.2482 |
|  |  | 17.00 | -1.04663 | 1.16360 | .946 | -4.4021 | 2.3088 |
|  | 15.00 | 12.00 | -.35502 | 1.07738 | .999 | -3.4619 | 2.7518 |
|  |  | 13.00 | .15874 | 1.11494 | 1.000 | -3.0564 | 3.3739 |
|  |  | 14.00 | -2.19205 | 1.12939 | .381 | -5.4489 | 1.0648 |
|  |  | 16.00 | -2.03143 | .96425 | .289 | -4.8121 | .7492 |
|  |  | 17.00 | -3.23869^*^ | 1.06647 | .033 | -6.3141 | -.1633 |
|  | 16.00 | 12.00 | 1.67641 | 1.01568 | .566 | -1.2525 | 4.6053 |
|  |  | 13.00 | 2.19017 | 1.05544 | .305 | -.8534 | 5.2337 |
|  |  | 14.00 | -.16062 | 1.07069 | 1.000 | -3.2482 | 2.9269 |
|  |  | 15.00 | 2.03143 | .96425 | .289 | -.7492 | 4.8121 |
|  |  | 17.00 | -1.20725 | 1.00410 | .835 | -4.1028 | 1.6883 |
|  | 17.00 | 12.00 | 2.88366 | 1.11318 | .105 | -.3264 | 6.0938 |
|  |  | 13.00 | 3.39742^*^ | 1.14957 | .041 | .0824 | 6.7125 |
|  |  | 14.00 | 1.04663 | 1.16360 | .946 | -2.3088 | 4.4021 |
|  |  | 15.00 | 3.23869^*^ | 1.06647 | .033 | .1633 | 6.3141 |
|  |  | 16.00 | 1.20725 | 1.00410 | .835 | -1.6883 | 4.1028 |
| FM (%) | 12.00 | 13.00 | 1.05635 | 1.47500 | .980 | -3.1971 | 5.3098 |
|  |  | 14.00 | -.50629 | 1.49268 | .999 | -4.8108 | 3.7982 |
|  |  | 15.00 | .45720 | 1.37030 | .999 | -3.4943 | 4.4087 |
|  |  | 16.00 | -2.20929 | 1.29183 | .527 | -5.9345 | 1.5160 |
|  |  | 17.00 | -2.15057 | 1.41584 | .653 | -6.2334 | 1.9323 |
|  | 13.00 | 12.00 | -1.05635 | 1.47500 | .980 | -5.3098 | 3.1971 |
|  |  | 14.00 | -1.56265 | 1.53665 | .912 | -5.9939 | 2.8686 |
|  |  | 15.00 | -.59916 | 1.41807 | .998 | -4.6885 | 3.4901 |
|  |  | 16.00 | -3.26564 | 1.34239 | .151 | -7.1367 | .6054 |
|  |  | 17.00 | -3.20692 | 1.46212 | .247 | -7.4233 | 1.0094 |
|  | 14.00 | 12.00 | .50629 | 1.49268 | .999 | -3.7982 | 4.8108 |
|  |  | 13.00 | 1.56265 | 1.53665 | .912 | -2.8686 | 5.9939 |
|  |  | 15.00 | .96349 | 1.43645 | .985 | -3.1788 | 5.1058 |
|  |  | 16.00 | -1.70299 | 1.36180 | .811 | -5.6300 | 2.2240 |
|  |  | 17.00 | -1.64428 | 1.47996 | .876 | -5.9121 | 2.6235 |
|  | 15.00 | 12.00 | -.45720 | 1.37030 | .999 | -4.4087 | 3.4943 |
|  |  | 13.00 | .59916 | 1.41807 | .998 | -3.4901 | 4.6885 |
|  |  | 14.00 | -.96349 | 1.43645 | .985 | -5.1058 | 3.1788 |
|  |  | 16.00 | -2.66648 | 1.22642 | .256 | -6.2031 | .8702 |
|  |  | 17.00 | -2.60777 | 1.35643 | .392 | -6.5193 | 1.3038 |
|  | 16.00 | 12.00 | 2.20929 | 1.29183 | .527 | -1.5160 | 5.9345 |
|  |  | 13.00 | 3.26564 | 1.34239 | .151 | -.6054 | 7.1367 |
|  |  | 14.00 | 1.70299 | 1.36180 | .811 | -2.2240 | 5.6300 |
|  |  | 15.00 | 2.66648 | 1.22642 | .256 | -.8702 | 6.2031 |
|  |  | 17.00 | .05872 | 1.27710 | 1.000 | -3.6241 | 3.7415 |
|  | 17.00 | 12.00 | 2.15057 | 1.41584 | .653 | -1.9323 | 6.2334 |
|  |  | 13.00 | 3.20692 | 1.46212 | .247 | -1.0094 | 7.4233 |
|  |  | 14.00 | 1.64428 | 1.47996 | .876 | -2.6235 | 5.9121 |
|  |  | 15.00 | 2.60777 | 1.35643 | .392 | -1.3038 | 6.5193 |
|  |  | 16.00 | -.05872 | 1.27710 | 1.000 | -3.7415 | 3.6241 |
| FM (kg) | 12.00 | 13.00 | .63383 | 1.79998 | .999 | -4.5568 | 5.8245 |
|  |  | 14.00 | -1.89724 | 1.82156 | .903 | -7.1501 | 3.3556 |
|  |  | 15.00 | -.56592 | 1.67221 | .999 | -5.3881 | 4.2562 |
|  |  | 16.00 | -3.82025 | 1.57645 | .154 | -8.3663 | .7258 |
|  |  | 17.00 | -4.72973 | 1.72778 | .073 | -9.7122 | .2527 |
|  | 13.00 | 12.00 | -.63383 | 1.79998 | .999 | -5.8245 | 4.5568 |
|  |  | 14.00 | -2.53107 | 1.87522 | .757 | -7.9386 | 2.8765 |
|  |  | 15.00 | -1.19975 | 1.73050 | .982 | -6.1900 | 3.7905 |
|  |  | 16.00 | -4.45408 | 1.63815 | .077 | -9.1780 | .2699 |
|  |  | 17.00 | -5.36356^*^ | 1.78427 | .036 | -10.5089 | -.2183 |
|  | 14.00 | 12.00 | 1.89724 | 1.82156 | .903 | -3.3556 | 7.1501 |
|  |  | 13.00 | 2.53107 | 1.87522 | .757 | -2.8765 | 7.9386 |
|  |  | 15.00 | 1.33132 | 1.75294 | .974 | -3.7236 | 6.3863 |
|  |  | 16.00 | -1.92302 | 1.66183 | .856 | -6.7153 | 2.8692 |
|  |  | 17.00 | -2.83249 | 1.80603 | .621 | -8.0406 | 2.3756 |
|  | 15.00 | 12.00 | .56592 | 1.67221 | .999 | -4.2562 | 5.3881 |
|  |  | 13.00 | 1.19975 | 1.73050 | .982 | -3.7905 | 6.1900 |
|  |  | 14.00 | -1.33132 | 1.75294 | .974 | -6.3863 | 3.7236 |
|  |  | 16.00 | -3.25434 | 1.49663 | .255 | -7.5702 | 1.0615 |
|  |  | 17.00 | -4.16381 | 1.65528 | .126 | -8.9372 | .6095 |
|  | 16.00 | 12.00 | 3.82025 | 1.57645 | .154 | -.7258 | 8.3663 |
|  |  | 13.00 | 4.45408 | 1.63815 | .077 | -.2699 | 9.1780 |
|  |  | 14.00 | 1.92302 | 1.66183 | .856 | -2.8692 | 6.7153 |
|  |  | 15.00 | 3.25434 | 1.49663 | .255 | -1.0615 | 7.5702 |
|  |  | 17.00 | -.90948 | 1.55848 | .992 | -5.4037 | 3.5847 |
|  | 17.00 | 12.00 | 4.72973 | 1.72778 | .073 | -.2527 | 9.7122 |
|  |  | 13.00 | 5.36356^*^ | 1.78427 | .036 | .2183 | 10.5089 |
|  |  | 14.00 | 2.83249 | 1.80603 | .621 | -2.3756 | 8.0406 |
|  |  | 15.00 | 4.16381 | 1.65528 | .126 | -.6095 | 8.9372 |
|  |  | 16.00 | .90948 | 1.55848 | .992 | -3.5847 | 5.4037 |
| LM (kg) | 12.00 | 13.00 | -.70957 | 1.59963 | .998 | -5.3224 | 3.9033 |
|  |  | 14.00 | -2.91409 | 1.61880 | .469 | -7.5823 | 1.7541 |
|  |  | 15.00 | -1.74226 | 1.48608 | .849 | -6.0277 | 2.5432 |
|  |  | 16.00 | -3.42390 | 1.40098 | .148 | -7.4639 | .6161 |
|  |  | 17.00 | -5.00593^*^ | 1.53547 | .017 | -9.4338 | -.5781 |
|  | 13.00 | 12.00 | .70957 | 1.59963 | .998 | -3.9033 | 5.3224 |
|  |  | 14.00 | -2.20453 | 1.66649 | .772 | -7.0102 | 2.6012 |
|  |  | 15.00 | -1.03269 | 1.53789 | .985 | -5.4675 | 3.4021 |
|  |  | 16.00 | -2.71434 | 1.45581 | .428 | -6.9125 | 1.4838 |
|  |  | 17.00 | -4.29636 | 1.58566 | .079 | -8.8690 | .2762 |
|  | 14.00 | 12.00 | 2.91409 | 1.61880 | .469 | -1.7541 | 7.5823 |
|  |  | 13.00 | 2.20453 | 1.66649 | .772 | -2.6012 | 7.0102 |
|  |  | 15.00 | 1.17183 | 1.55782 | .975 | -3.3205 | 5.6641 |
|  |  | 16.00 | -.50981 | 1.47686 | .999 | -4.7686 | 3.7490 |
|  |  | 17.00 | -2.09184 | 1.60501 | .783 | -6.7202 | 2.5365 |
|  | 15.00 | 12.00 | 1.74226 | 1.48608 | .849 | -2.5432 | 6.0277 |
|  |  | 13.00 | 1.03269 | 1.53789 | .985 | -3.4021 | 5.4675 |
|  |  | 14.00 | -1.17183 | 1.55782 | .975 | -5.6641 | 3.3205 |
|  |  | 16.00 | -1.68164 | 1.33004 | .804 | -5.5171 | 2.1538 |
|  |  | 17.00 | -3.26367 | 1.47104 | .235 | -7.5057 | .9784 |
|  | 16.00 | 12.00 | 3.42390 | 1.40098 | .148 | -.6161 | 7.4639 |
|  |  | 13.00 | 2.71434 | 1.45581 | .428 | -1.4838 | 6.9125 |
|  |  | 14.00 | .50981 | 1.47686 | .999 | -3.7490 | 4.7686 |
|  |  | 15.00 | 1.68164 | 1.33004 | .804 | -2.1538 | 5.5171 |
|  |  | 17.00 | -1.58202 | 1.38501 | .863 | -5.5760 | 2.4119 |
|  | 17.00 | 12.00 | 5.00593^*^ | 1.53547 | .017 | .5781 | 9.4338 |
|  |  | 13.00 | 4.29636 | 1.58566 | .079 | -.2762 | 8.8690 |
|  |  | 14.00 | 2.09184 | 1.60501 | .783 | -2.5365 | 6.7202 |
|  |  | 15.00 | 3.26367 | 1.47104 | .235 | -.9784 | 7.5057 |
|  |  | 16.00 | 1.58202 | 1.38501 | .863 | -2.4119 | 5.5760 |
| ALM (kg) | 12.00 | 13.00 | -.30866 | .80879 | .999 | -2.6410 | 2.0237 |
|  |  | 14.00 | -.79510 | .81849 | .926 | -3.1554 | 1.5652 |
|  |  | 15.00 | -.75983 | .75138 | .914 | -2.9266 | 1.4069 |
|  |  | 16.00 | -1.75692 | .70835 | .136 | -3.7996 | .2858 |
|  |  | 17.00 | -1.75840 | .77635 | .215 | -3.9972 | .4804 |
|  | 13.00 | 12.00 | .30866 | .80879 | .999 | -2.0237 | 2.6410 |
|  |  | 14.00 | -.48644 | .84260 | .992 | -2.9163 | 1.9434 |
|  |  | 15.00 | -.45116 | .77757 | .992 | -2.6935 | 1.7911 |
|  |  | 16.00 | -1.44826 | .73608 | .366 | -3.5709 | .6744 |
|  |  | 17.00 | -1.44974 | .80173 | .463 | -3.7617 | .8622 |
|  | 14.00 | 12.00 | .79510 | .81849 | .926 | -1.5652 | 3.1554 |
|  |  | 13.00 | .48644 | .84260 | .992 | -1.9434 | 2.9163 |
|  |  | 15.00 | .03528 | .78765 | 1.000 | -2.2361 | 2.3066 |
|  |  | 16.00 | -.96182 | .74672 | .791 | -3.1151 | 1.1915 |
|  |  | 17.00 | -.96330 | .81151 | .843 | -3.3035 | 1.3769 |
|  | 15.00 | 12.00 | .75983 | .75138 | .914 | -1.4069 | 2.9266 |
|  |  | 13.00 | .45116 | .77757 | .992 | -1.7911 | 2.6935 |
|  |  | 14.00 | -.03528 | .78765 | 1.000 | -2.3066 | 2.2361 |
|  |  | 16.00 | -.99710 | .67249 | .676 | -2.9363 | .9422 |
|  |  | 17.00 | -.99858 | .74377 | .761 | -3.1434 | 1.1462 |
|  | 16.00 | 12.00 | 1.75692 | .70835 | .136 | -.2858 | 3.7996 |
|  |  | 13.00 | 1.44826 | .73608 | .366 | -.6744 | 3.5709 |
|  |  | 14.00 | .96182 | .74672 | .791 | -1.1915 | 3.1151 |
|  |  | 15.00 | .99710 | .67249 | .676 | -.9422 | 2.9363 |
|  |  | 17.00 | -.00148 | .70028 | 1.000 | -2.0209 | 2.0179 |
|  | 17.00 | 12.00 | 1.75840 | .77635 | .215 | -.4804 | 3.9972 |
|  |  | 13.00 | 1.44974 | .80173 | .463 | -.8622 | 3.7617 |
|  |  | 14.00 | .96330 | .81151 | .843 | -1.3769 | 3.3035 |
|  |  | 15.00 | .99858 | .74377 | .761 | -1.1462 | 3.1434 |
|  |  | 16.00 | .00148 | .70028 | 1.000 | -2.0179 | 2.0209 |
| LMI (kg/m²) | 12.00 | 13.00 | .11736 | .51965 | 1.000 | -1.3812 | 1.6159 |
|  |  | 14.00 | -.38944 | .52588 | .977 | -1.9059 | 1.1270 |
|  |  | 15.00 | .23692 | .48276 | .996 | -1.1552 | 1.6291 |
|  |  | 16.00 | -.50503 | .45511 | .877 | -1.8174 | .8074 |
|  |  | 17.00 | -1.24863 | .49881 | .129 | -2.6870 | .1898 |
|  | 13.00 | 12.00 | -.11736 | .51965 | 1.000 | -1.6159 | 1.3812 |
|  |  | 14.00 | -.50680 | .54137 | .937 | -2.0679 | 1.0544 |
|  |  | 15.00 | .11957 | .49959 | 1.000 | -1.3211 | 1.5602 |
|  |  | 16.00 | -.62239 | .47293 | .776 | -1.9862 | .7414 |
|  |  | 17.00 | -1.36599 | .51511 | .091 | -2.8514 | .1194 |
|  | 14.00 | 12.00 | .38944 | .52588 | .977 | -1.1270 | 1.9059 |
|  |  | 13.00 | .50680 | .54137 | .937 | -1.0544 | 2.0679 |
|  |  | 15.00 | .62636 | .50607 | .818 | -.8330 | 2.0857 |
|  |  | 16.00 | -.11559 | .47977 | 1.000 | -1.4991 | 1.2679 |
|  |  | 17.00 | -.85919 | .52140 | .568 | -2.3627 | .6444 |
|  | 15.00 | 12.00 | -.23692 | .48276 | .996 | -1.6291 | 1.1552 |
|  |  | 13.00 | -.11957 | .49959 | 1.000 | -1.5602 | 1.3211 |
|  |  | 14.00 | -.62636 | .50607 | .818 | -2.0857 | .8330 |
|  |  | 16.00 | -.74195 | .43207 | .522 | -1.9879 | .5040 |
|  |  | 17.00 | -1.48556^*^ | .47787 | .026 | -2.8636 | -.1075 |
|  | 16.00 | 12.00 | .50503 | .45511 | .877 | -.8074 | 1.8174 |
|  |  | 13.00 | .62239 | .47293 | .776 | -.7414 | 1.9862 |
|  |  | 14.00 | .11559 | .47977 | 1.000 | -1.2679 | 1.4991 |
|  |  | 15.00 | .74195 | .43207 | .522 | -.5040 | 1.9879 |
|  |  | 17.00 | -.74360 | .44993 | .565 | -2.0411 | .5539 |
|  | 17.00 | 12.00 | 1.24863 | .49881 | .129 | -.1898 | 2.6870 |
|  |  | 13.00 | 1.36599 | .51511 | .091 | -.1194 | 2.8514 |
|  |  | 14.00 | .85919 | .52140 | .568 | -.6444 | 2.3627 |
|  |  | 15.00 | 1.48556^*^ | .47787 | .026 | .1075 | 2.8636 |
|  |  | 16.00 | .74360 | .44993 | .565 | -.5539 | 2.0411 |
