# Supplementary material for: Lean mass reference curves in adolescents using dual-energy x-ray absorptiometry (DXA)
Source: PLoS One. 2020 Feb 6;15(2):e0228646. doi: 10.1371/journal.pone.0228646 (PMC7004364; doi:10.1371/journal.pone.0228646)
Supplement: S3 Table — (DOCX) [file pone.0228646.s003.docx]

**SUPPLEMENTARY MATERIAL**

| **Supplementary Table 3 - LMS for boys** | | | | | | | | | |
| --- | --- | --- | --- | --- | --- | --- | --- | --- | --- |
|  |  |  |  |  |  |  |  |  |  |
| **Appendicular Lean Mass (kg) - Boys** | | | | | | | | | |
| **Age** | **L** | **S** | **3rd** | **10th** | **25th** | **50th (M)** | **75th** | **90th** | **97th** |
| 12.00 | -0.53 | 0.21 | 9.46 | 10.51 | 11.77 | 13.18 | 15.56 | 17.90 | 20.78 |
| 13.00 | 0.10 | 0.20 | 11.70 | 13.22 | 14.93 | 16.72 | 19.47 | 21.89 | 24.54 |
| 14.00 | 0.63 | 0.18 | 13.66 | 15.60 | 17.66 | 20.02 | 22.58 | 24.94 | 27.35 |
| 15.00 | 0.96 | 0.16 | 14.86 | 16.90 | 18.98 | 21.05 | 23.62 | 25.73 | 27.81 |
| 16.00 | 1.22 | 0.14 | 15.91 | 17.96 | 19.97 | 22.28 | 24.31 | 26.20 | 28.04 |
| 17.00 | 1.41 | 0.13 | 17.26 | 19.27 | 21.22 | 22.90 | 25.32 | 27.08 | 28.76 |
|  |  |  |  |  |  |  |  |  |  |
| **Lean Mass Index (kg/m²) - Boys** | | | | | | | | | |
| **Age** | **L** | **S** | **3rd** | **10th** | **25th** | **50th (M)** | **75th** | **90th** | **97th** |
| 12.00 | 0.15 | 0.13 | 10.63 | 11.55 | 12.55 | 13.51 | 15.04 | 16.29 | 17.61 |
| 13.00 | 0.49 | 0.13 | 11.48 | 12.50 | 13.58 | 14.24 | 16.14 | 17.37 | 18.63 |
| 14.00 | 0.86 | 0.12 | 12.38 | 13.51 | 14.67 | 15.93 | 17.28 | 18.48 | 19.67 |
| 15.00 | 1.03 | 0.11 | 12.82 | 13.93 | 15.04 | 16.24 | 17.51 | 18.62 | 19.71 |
| 16.00 | 1.21 | 0.10 | 13.12 | 14.20 | 15.27 | 16.27 | 17.59 | 18.61 | 19.61 |
| 17.00 | 1.47 | 0.10 | 13.93 | 15.01 | 16.08 | 17.49 | 18.34 | 19.31 | 20.25 |
|  |  |  |  |  |  |  |  |  |  |
| **Fat mass (kg) - Boys** | | | | | | | | | |
| **Age** | **L** | **S** | **3rd** | **10th** | **25th** | **50th (M)** | **75th** | **90th** | **97th** |
| 12.00 | -0.74 | 0.38 | 6.18 | 7.25 | 8.70 | 11.24 | 14.69 | 20.31 | 30.92 |
| 13.00 | -1.01 | 0.33 | 6.89 | 7.84 | 9.11 | 11.46 | 14.27 | 19.17 | 29.04 |
| 14.00 | -1.21 | 0.28 | 7.27 | 8.11 | 9.22 | 11.17 | 13.58 | 17.58 | 25.50 |
| 15.00 | -1.28 | 0.25 | 7.58 | 8.38 | 9.42 | 10.70 | 13.34 | 16.75 | 23.00 |
| 16.00 | -1.30 | 0.25 | 7.49 | 8.27 | 9.27 | 10.64 | 13.00 | 16.20 | 21.96 |
| 17.00 | -1.34 | 0.25 | 7.98 | 8.82 | 9.90 | 11.77 | 14.05 | 17.74 | 24.83 |
|  |  |  |  |  |  |  |  |  |  |
| **Lean Mass (kg) - Boys** | | | | | | | | | |
| **Age** | **L** | **S** | **3rd** | **10th** | **25th** | **50th (M)** | **75th** | **90th** | **97th** |
| 12.00 | -0.21 | 0.20 | 21.95 | 24.57 | 27.64 | 31.42 | 36.27 | 41.20 | 46.88 |
| 13.00 | 0.38 | 0.19 | 26.61 | 30.18 | 34.08 | 38.49 | 43.84 | 48.74 | 53.89 |
| 14.00 | 0.79 | 0.17 | 31.38 | 35.62 | 40.03 | 44.59 | 50.18 | 54.90 | 59.64 |
| 15.00 | 0.89 | 0.14 | 34.84 | 38.86 | 42.97 | 46.98 | 52.26 | 56.50 | 60.72 |
| 16.00 | 0.81 | 0.13 | 37.29 | 40.99 | 44.80 | 49.18 | 53.49 | 57.50 | 61.50 |
| 17.00 | 0.56 | 0.12 | 40.56 | 44.12 | 47.86 | 52.44 | 56.65 | 60.82 | 65.06 |

Appendicular lean mass (ALM); fat mass (FM); lean mass (LM); lean mass index (LMI).
