# Supplementary material for: Lean mass reference curves in adolescents using dual-energy x-ray absorptiometry (DXA)
Source: PLoS One. 2020 Feb 6;15(2):e0228646. doi: 10.1371/journal.pone.0228646 (PMC7004364; doi:10.1371/journal.pone.0228646)
Supplement: S4 Table — (DOCX) [file pone.0228646.s004.docx]

**SUPPLEMENTARY MATERIAL**

| **Supplementary Table 4 - LMS for girls** | | | | | | | | | |
| --- | --- | --- | --- | --- | --- | --- | --- | --- | --- |
|  |  |  |  |  |  |  |  |  |  |
| **Appendicular Lean Mass (kg) - Girls** | | | | | | | | | |
| **Age** | **L** | **S** | **3rd** | **10th** | **25th** | **50th (M)** | **75th** | **90th** | **97th** |
| 12.00 | -0.04 | 0.16 | 10.27 | 11.31 | 12.47 | 13.55 | 15.52 | 17.14 | 18.91 |
| 13.00 | -0.30 | 0.18 | 10.34 | 11.39 | 12.61 | 14.35 | 15.98 | 17.88 | 20.06 |
| 14.00 | -0.39 | 0.19 | 10.27 | 11.35 | 12.62 | 14.13 | 16.23 | 18.33 | 20.80 |
| 15.00 | -0.31 | 0.19 | 10.55 | 11.68 | 13.00 | 14.63 | 16.70 | 18.82 | 21.27 |
| 16.00 | -0.04 | 0.18 | 11.05 | 12.30 | 13.71 | 15.12 | 17.49 | 19.53 | 21.79 |
| 17.00 | 0.59 | 0.17 | 11.06 | 12.49 | 14.01 | 15.58 | 17.63 | 19.37 | 21.15 |
|  |  |  |  |  |  |  |  |  |  |
| **Lean Mass Index (kg/m²) - Girls** | | | | | | | | | |
| **Age** | **L** | **S** | **3rd** | **10th** | **25th** | **50th (M)** | **75th** | **90th** | **97th** |
| 12.00 | -0.35 | 0.12 | 11.13 | 11.88 | 12.72 | 13.64 | 14.89 | 16.02 | 17.26 |
| 13.00 | -0.83 | 0.12 | 11.03 | 11.77 | 12.60 | 13.76 | 14.92 | 16.23 | 17.75 |
| 14.00 | -0.77 | 0.13 | 10.97 | 11.74 | 12.61 | 14.43 | 15.04 | 16.42 | 18.01 |
| 15.00 | -0.33 | 0.13 | 10.81 | 11.61 | 12.51 | 13.56 | 14.87 | 16.12 | 17.50 |
| 16.00 | 0.02 | 0.12 | 11.31 | 12.17 | 13.12 | 14.40 | 15.47 | 16.66 | 17.93 |
| 17.00 | 0.12 | 0.12 | 12.03 | 12.92 | 13.89 | 15.24 | 16.27 | 17.45 | 18.69 |
|  |  |  |  |  |  |  |  |  |  |
| **Fat mass (kg) - Girls** | | | | | | | | | |
| **Age** | **L** | **S** | **3rd** | **10th** | **25th** | **50th (M)** | **75th** | **90th** | **97th** |
| 12.00 | 0.07 | 0.34 | 8.71 | 10.73 | 13.22 | 16.24 | 20.78 | 25.36 | 30.78 |
| 13.00 | 0.03 | 0.31 | 9.19 | 11.13 | 13.49 | 16.93 | 20.64 | 24.95 | 30.06 |
| 14.00 | 0.01 | 0.31 | 10.11 | 12.16 | 14.65 | 17.90 | 22.11 | 26.60 | 31.90 |
| 15.00 | 0.08 | 0.31 | 9.99 | 12.09 | 14.63 | 16.26 | 22.12 | 26.53 | 31.66 |
| 16.00 | 0.10 | 0.31 | 11.05 | 13.45 | 16.35 | 21.16 | 24.88 | 29.88 | 35.70 |
| 17.00 | -0.05 | 0.31 | 12.11 | 14.51 | 17.46 | 20.48 | 26.51 | 32.08 | 38.79 |
|  |  |  |  |  |  |  |  |  |  |
| **Lean Mass (kg) - Girls** | | | | | | | | | |
| **Age** | **L** | **S** | **3rd** | **10th** | **25th** | **50th (M)** | **75th** | **90th** | **97th** |
| 12.00 | 0.51 | 0.16 | 24.27 | 27.08 | 30.07 | 32.86 | 37.27 | 40.75 | 44.34 |
| 13.00 | -0.46 | 0.15 | 26.36 | 28.56 | 31.08 | 34.12 | 38.00 | 41.86 | 46.27 |
| 14.00 | -1.16 | 0.14 | 27.69 | 29.64 | 31.96 | 35.05 | 38.82 | 43.09 | 48.44 |
| 15.00 | -1.15 | 0.14 | 27.92 | 29.86 | 32.15 | 35.49 | 38.90 | 43.06 | 48.24 |
| 16.00 | -0.73 | 0.14 | 28.58 | 30.73 | 33.20 | 36.36 | 40.14 | 44.13 | 48.78 |
| 17.00 | -0.09 | 0.14 | 29.70 | 32.21 | 34.99 | 38.58 | 42.13 | 45.85 | 49.88 |

Appendicular lean mass (ALM); fat mass (FM); lean mass (LM); lean mass index (LMI).
