# Supplementary material for: Lean mass reference curves in adolescents using dual-energy x-ray absorptiometry (DXA)
Source: PLoS One. 2020 Feb 6;15(2):e0228646. doi: 10.1371/journal.pone.0228646 (PMC7004364; doi:10.1371/journal.pone.0228646)
Supplement: S5 Table — Appendicular Lean Mass (ALM). Lean Mass Index (LMI) and Fat Mass (FM). (DOCX) [file pone.0228646.s005.docx]

**SUPPLEMENTARY MATERIAL**

| **Supplementary Table 5 - Difference between sex for Lean Mass (LM). Appendicular Lean Mass (ALM). Lean Mass Index (LMI) and Fat Mass (FM)** | | | | | | | | | | | | |
| --- | --- | --- | --- | --- | --- | --- | --- | --- | --- | --- | --- | --- |
| **Age** | **12** | | **13** | | **14** | | **15** | | **16** | | **17** | |
|  | **t** | **p** | **t** | **p** | **t** | **p** | **t** | **p** | **t** | **p** | **t** | **p** |
| **LM** | -0.974 | 0.334 | 2.559 | 0.013 | 4.427 | 0.000 | 10.251 | 0.000 | 9.814 | 0.000 | 8.888 | 0.000 |
| **ALM** | -0.452 | 0.653 | 3.464 | 0.001 | 5.819 | 0.000 | 10.324 | 0.000 | 9.832 | 0.000 | 10.439 | 0.000 |
| **LMI** | 0.013 | 0.990 | 2.210 | 0.031 | 3.471 | 0.001 | 7.438 | 0.000 | 5.692 | 0.000 | 5.115 | 0.000 |
| **FM** | -2.492 | 0.016 | -3.206 | 0.002 | -4.901 | 0.000 | -6.212 | 0.000 | -9.510 | 0.000 | -6.509 | 0.000 |
